# The Expression/Methylation Profile of Adipogenic and Inflammatory Transcription Factors in Adipose Tissue Are Linked to Obesity-Related Colorectal Cancer

**DOI:** 10.3390/cancers11111629

**Published:** 2019-10-24

**Authors:** Hatim Boughanem, Amanda Cabrera-Mulero, Pablo Hernández-Alonso, Borja Bandera-Merchán, Alberto Tinahones, Francisco José Tinahones, Sonsoles Morcillo, Manuel Macias-Gonzalez

**Affiliations:** 1 Biomedical Research Institute of Malaga (IBIMA), Faculty of Science, University of Malaga, 29010 Málaga, Spain; 2 Department of Endocrinology and Nutrition, Virgen de la Victoria University Hospital, University of Malaga (IBIMA), 29010 Málaga, Spain; 3 CIBEROBN (CIBER in Physiopathology of Obesity and Nutrition CB06/03/0018), Instituto de Salud Carlos III, 28029 Madrid, Spain; 4 Human Nutrition Unit, Faculty of Medicine and Health Sciences, Sant Joan Hospital, Institut d’Investigació Sanitària Pere Virgili, Rovira i Virgili University, 43201 Reus, Spain

**Keywords:** C/EBP-α, PPAR-γ, PGC-1α, NF-κB, obesity, visceral adipose tissue, colorectal cancer

## Abstract

Obesity is well accepted as crucial risk factor that plays a critical role in the initiation and progression of colorectal cancer (CRC). More specifically, visceral adipose tissue (VAT) in people with obesity could produce chronic inflammation and an altered profile expression of key transcription factors that promote a favorable microenvironment to colorectal carcinogenesis. For this, the aim of this study was to explore the relationship between adipogenic and inflammatory transcription factors in VAT from nonobese, obese, and/or CRC patients. To test this idea, we studied the expression and methylation of CCAAT-enhancer binding protein type alpha (C/EBP-α), peroxisome proliferator-activated receptor gamma (PPAR-γ), peroxisome proliferator-activated receptor gamma coactivator 1-α (PGC-1α) and nuclear factor κ-light-chain-enhancer of activated B cells (NF-κB) in VAT from non-obese control, non-obese CRC subjects, overweight/obese control, and overweight/obese CRC patients and their correlation with anthropometric and biochemical variables. We found decreased expression of C/EBP-α in overweight/obese CRC patients in comparison with overweight/obese control subjects. PGC-1α and NF-κB were overexpressed in CRC patients independently of the BMI. NF-κB promoter was hypomethylated in overweight/obese CRC patients when compared to overweight/obese control individuals. In addition, multiple significant correlations between expression, methylation, and biochemical parameters were found. Finally, linear regression analysis showed that the expression of C/EBP-α and NF-κB and that NF-κB methylation were associated with CRC and able to explain up to 55% of CRC variability. Our results suggest that visceral adipose tissue may be a key factor in tumor development and inflammatory state. We propose C/EBP-α, PGC-1α and NF-κB to be interesting candidates as potential biomarkers in adipose tissue for CRC patients.

## 1. Introduction

Colorectal cancer (CRC) is considered the most important public health concern, which constitutes the third most commonly occurring cancer worldwide in both men and women [1]. Incidence and mortality trends of CRC have significantly increased in the last few years, although there are more new existing tools and biomarkers for early diagnostic [2,3]. The etiology of CRC is multifactorial and complex, resulting from a combination of environmental, genetic, and epigenetic factors, but obesity is well accepted as a crucial risk factor that plays a critical role in the initiation and progression of CRC [4]. Indeed, it is well known that visceral adipose tissue (VAT) in people with obesity could produce chronic inflammation and an altered profile expression of key transcription factors that promote a favorable microenvironment to colorectal carcinogenesis [5,6].

Among them, CCAAT-enhancer binding protein type alpha (C/EBP-α) and peroxisome proliferator-activated receptor gamma (PPAR-γ) are two transcription factors that orchestrate the adipogenesis process but are also involved in carcinogenesis [7,8]. In fact, the expression of C/EBP-α has been described in different studies. While some studies introduced a downregulation of C/EBP-α in primary CRC tumors in comparison with normal colon cells CRC [9,10], another study pointed to an upregulation of C/EBP-α in CRC tumors. This study also showed that C/EBP-α expression enhances CRC cells migration and invasion in vitro as well as metastasis in vivo [11]. As for PPAR-γ expression, a study reported that the expression profile of PPAR-γ was significantly lower in primary tumors when compared to normal colon for CRC patients [12]. However, others studies did not find significant differences between tumor and margins tissues [13]. The PPAR-γ expression was also not correlated with BMI and grade of dysplasia [14]. However, to date, there is a lack of studies of the expression profile, methylation, and functional significance in VAT of C/EBP-α and PPAR-γ in obesity-related CRC.

The master regulator of energy metabolism and mitochondrial biogenesis, peroxisome proliferator-activated receptor gamma coactivator 1-α (PPARGC1A or PGC-1α), integrates and coordinates the activity of other transcription factors, such as PPAR-γ. Previous studies showed a biphasic role of PGC-1α in cancer, acting both as tumor suppressor and tumor promoter gene [15]. Several studies indicate an altered expression profile of PGC-1α in CRC risk. Most studies showed that overexpression of PGC-1α increases CRC risk, while other studies showed that lower expression levels could increase CRC risk [16]. A study reported that downregulation of PGC-1α inhibited the migration and invasion in LoVo cells. PGC-1α expression was also higher in the colorectal cancer tissues than that in para-cancerous tissues, and its expression in the invading front area was higher than that in the tumor center area [17]. In contrast, overexpression of PGC-1α enhances cell proliferation and tumorigenesis of HEK293 cells [18]. PGC-1α expression was also correlated with nodal metastasis [19]. Although there are not widely accepted mechanisms to explain the role of PGC-1α in human CRC, it might be connected with an increased cancer risk in the context of obesity [20].

On the other hand, a number of studies discovered the relationship between obesity, CRC, and low-grade inflammation by aberrant activation of nuclear factor κ-light-chain-enhancer of activated B cells (NF-κB) [6,21]. NF-κB is a transcription factor that is considered the master regulator of inflammation processes. NF-κB has a paradoxical effect in cancer, since it is responsible for dual acute and chronic inflammation. While acute inflammation inhibits cancer growth, the chronic inflammation implicates tumor development [12]. However, a study showed that NF-κB expression and methylation were altered in adipose tissue of CRC patients, which suggests a possible mediation effect of VAT in CRC development by modifying adipose tissue DNA methylation and by promoting inflammation [6]. This study showed an increased expression of NF-κB in VAT from CRC patients and low methylation status in the NF-κB promoter in comparison with non-CRC patients. Another study conducted in peripheral blood showed that the mRNA expression of NF-κB was significantly lower in both the T2 and T3 stages of CRC as compared to the T4 stage [22].

In any case, epigenetic modifications, including DNA methylation, are supported to be one of the links between obesity and CRC. Several large-scale epigenetic and methylome analyses reported that an altered DNA methylation profile in CRC was associated to the body mass index (BMI) [23]. Indeed, there are more and more studies that provide an epigenetic signature specific for each tumor and associated with obesity [23]. Other studies reported evidences of the role of DNA methylation of adipogenic and inflammatory gene promoters on obesity and cancer [4]. For instance, hypermethylation of the C/EBP-α promoter was associated with downregulation of C/EBP-α expression in several cancer [24]. In the same line, PPAR-γ promoter methylation was also related to poor prognosis in CRC [25]. PGC-1α methylation was linked to cancer and is considered an early biomarker of cancer risk because it may be a possible link between telomere shortening and mitochondrial dysfunction [26]. Finally, NF-κB methylation was lower in CRC patients when compared to the non-cancer group, and a negative trend was observed between NF-κB mRNA levels and NF-κB methylation [6].

Therefore, in our study, we hypothesized that a dysfunctional adipose tissue could lead to an altered adipogenic and inflammatory expression profile in the context of obesity and CRC, which could increase the risk of CRC. Thus, the aim of this study was to test the expression profile and methylation status of C/EBP-α, PPAR-γ, PGC-1α, and NF-κB in VAT from nonobese and obese individuals without/with CRC. In addition, we also investigated the association between gene expression and methylation status of these genes with anthropometric and biochemical variables to clarify the correlation between BMI and CRC. Finally, we studied under linear regression model the effect of these correlated genes, including BMI as an additional factor to predict the risk of CRC.

## 2. Results

### 2.1. Characteristics of the Study Population

Anthropometric and biochemical variables of nonobese subjects (N = 53), nonobese CRC patients (N = 27), overweight/obese subjects (N = 81), and overweight/obese CRC patients (N = 58) are summarized in Table 1. Nonobese CRC patients had significantly increased insulin levels and significantly decreased high density lipoprotein (HDL) when compared to nonobese subjects (*p* < 0.05). In addition, there were significant differences in sex and age between both groups (*p* < 0.05). The overweight/obese CRC group had significantly greater triglycerides levels as compared to overweight/obese subjects (*p* < 0.05). Moreover, total cholesterol, HDL, low density lipoprotein (LDL), and insulin were significantly decreased in overweight/obese CRC patients in comparison with overweight/obese subjects (*p* < 0.05). Sex and age were also significantly different between overweight/obese control and overweight/obese CRC patients (*p* < 0.05).

### 2.2. Expression and Methylation Profile of C/EBP-α and PPAR-γ in VAT in Obesity-Related CRC

**Figure 1:**
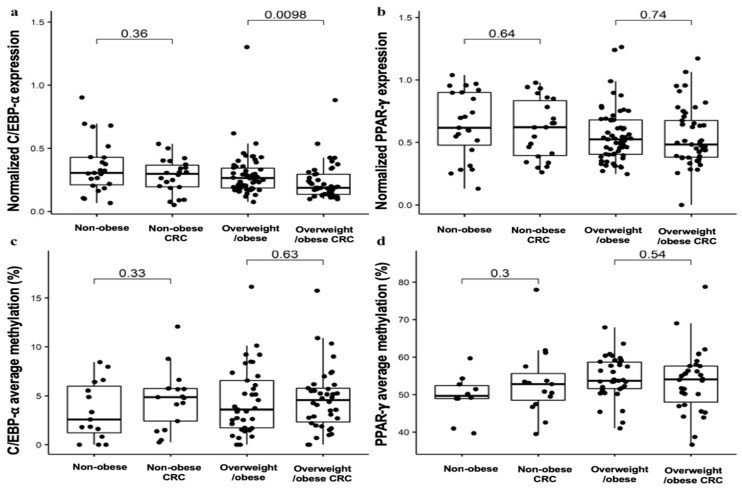
Gene expression and methylation analyses of CCAAT-enhancer binding protein type alpha (C/EBP-α) and peroxisome proliferator-activated receptor gamma (PPAR-γ) in visceral adipose tissue (VAT): Quantitative RT-PCR of C/EBP-α (**a**) and PPAR-γ (**b**) expression analyses and methylation analyses at specific CpG dinucleotides for C/EBP-α (**c**) and PPAR-γ (**d**) promoters was used to compare the nonobese control (N = 53), nonobese CRC (N = 27), overweight/obese control (N = 81), and overweight/obese CRC groups (N = 58). The mRNA expression of C/EBP-α and PPAR-γ were normalized to Peptidylprolyl Isomerase A (PPIA) expression. The results are given as the mRNA relative mean expression ± SD and methylation average ± SD. Significant differences between the means of the different groups of subjects was performed according to the Wilcoxon test (*p* < 0.05). Abbreviations: C/EBP-α: CCAAT/enhancer-binding protein type alpha; PPAR-γ2: peroxisome proliferator-activated receptor gamma; VAT: Visceral adipose tissue.

The mean levels of C/EBP-α gene expression in VAT in the groups are shown in Figure 1a, and the C/EBP-α average methylation (%) in VAT are presented in Figure 1c. C/EBP-α gene expression in overweight/obese CRC patients was significantly decreased as compared to overweight/obese subjects (*p* < 0.05) (Figure 1a). However, no significant differences were found on methylation status of C/EBP-α promoter between groups (Figure 1c). We next investigated the gene expression profile and methylation status of PPAR-γ in VAT, which are shown in Figure 1b,d. Our analysis showed that no significant difference was observed between nonobese subjects and nonobese CRC patients or between overweight/obese subjects and overweight/obese CRC patients (Figure 1b,d). For more analysis between all groups, see Figure S1.

**Figure 2:**
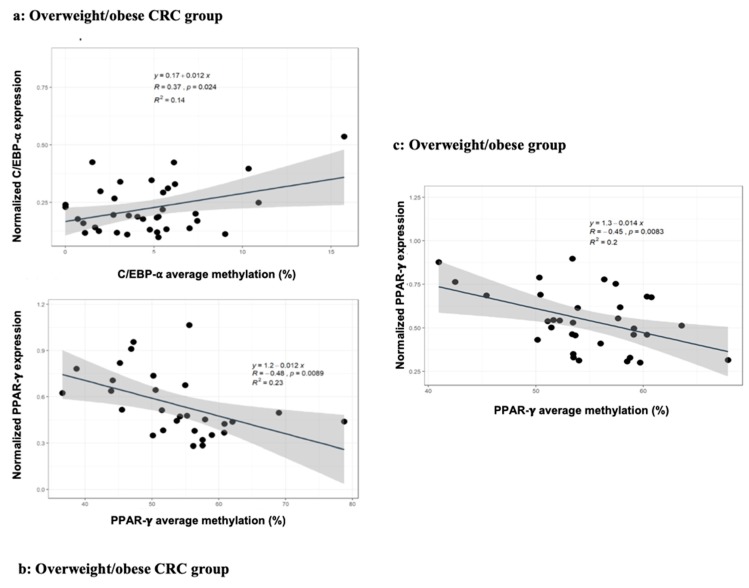
Correlation between gene expression and methylation analyses of C/EBP-α and PPAR-γ in VAT: Pearson’s correlation between gene expression and methylation analyses of C/EBP-α in VAT from the overweight/obese CRC groups (N = 58) (**a**) and of PPAR-γ in VAT from the overweight/obese control (N = 81) (**b**) and overweight/obese CRC groups (N = 58) (**c**) (*p* < 0.05). Abbreviations: C/EBP-α: CCAAT/enhancer-binding protein type alpha; PPAR-γ2: peroxisome proliferator-activated receptor gamma; VAT: Visceral adipose tissue.

To verify any association between the gene expression and methylation status of C/EBP-α and PPAR-γ in the populations study, a Pearson’s correlation analysis was performed in each group (Figure 2). A significant positive correlation was found between gene expression and methylation status of C/EBP-α in overweight/obese CRC patients (r = 0.37; *p* = 0.02) (Figure 2a). In contrast, we found a significant negative correlation between gene expression and methylation average of PPAR-γ in overweight/obese CRC patients (r = −0.48; *p* = 0.00) (Figure 2b) and overweight/obese individuals (r = −0.45; *p* = 0.00) (Figure 2c).

### 2.3. Expression and Methylation Profile of PGC-1α and NF-κB in VAT in Obesity-Related CRC

**Figure 3:**
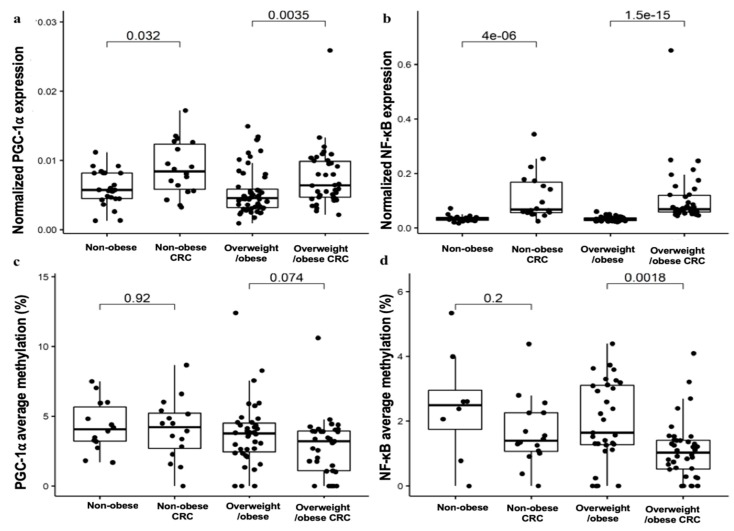
Gene expression and methylation analyses of PGC-1α and NF-κB in VAT: Quantitative RT-PCR of PGC-1α (**a**) and NF-κB (**b**) expression analyses and methylation analyses at specific CpG dinucleotides for PGC-1α (**c**) and NF-κB (**d**) promoters was used to compare the nonobese control (N = 53), nonobese CRC (N = 27), overweight/obese control (N = 81), and overweight/obese CRC groups (N = 58). The mRNA expression of PGC-1α and NF-κB was normalized to the PPIA expression. The results are given as the mRNA relative mean expression ± SD and methylation average ± SD. Significant differences between the means of the different groups of subjects was performed according to the Wilcoxon test (*p* < 0.05). Abbreviations: PGC-1α: peroxisome proliferator-activated receptor gamma coactivator 1-α; NF-κB: nuclear factor κ-light-chain-enhancer of activated B cells; VAT: Visceral adipose tissue.

Here, we tested the expression profile and methylation analysis of PGC-1α as the central regulator of metabolism energy and of NF-κB as the central regulator of inflammation in VAT. Our results showed that PGC-1α gene expression in VAT was significantly increased in nonobese CRC and overweight/obese CRC as compared to nonobese and overweight/obese subjects, respectively (*p* < 0.05) (Figure 3a). However, the DNA methylation status of the PGC-1α promoter did not show significant difference between both groups (Figure 3c). As for the expression profile of NF-κB in VAT, we observed the same trend of PGC-1α expression profile (Figure 3). Nonobese CRC and overweight/obese CRC patients showed higher expression levels of NF-κB in comparison with nonobese and overweight/obese subjects, respectively (*p* < 0.05) (Figure 3b). Moreover, DNA methylation status of the NF-κB promoter in VAT was significantly decreased in overweight/obese CRC as compared to overweight/obese subjects (*p* < 0.05) (Figure 3d). For more analysis between all groups, see Figure S2.

### 2.4. Correlation and Regression Analyses Between Gene Expression, Methylation Status, and Biochemical and Anthropometric Data

**Figure 4:**
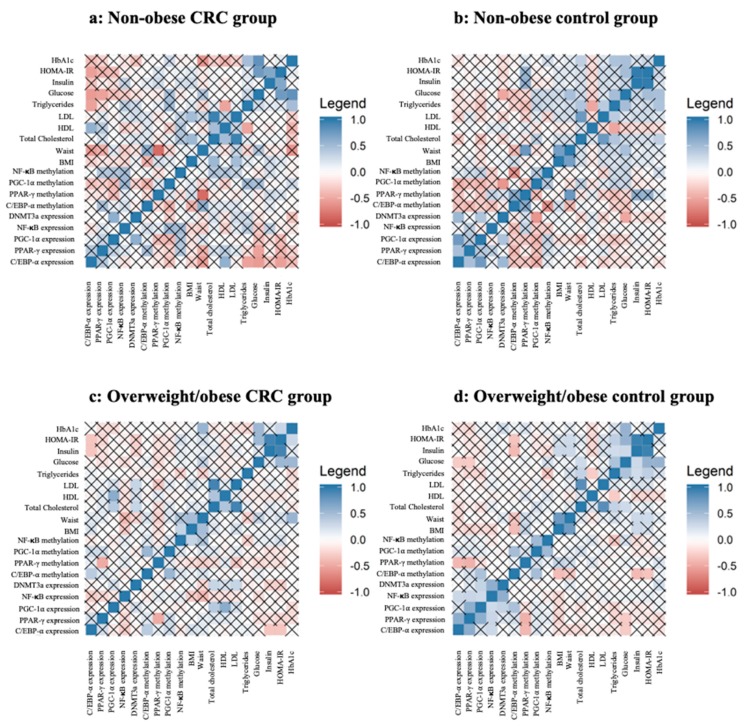
Correlation between gene expression and methylation analyses of C/EBP-α, PPAR-γ, PGC-1α, and NF-κB in VAT and anthropometric and biochemical data. Pearson’s correlation between Gene expression and methylation analyses of C/EBP-α, PPAR-γ, PGC-1α, and NF-κB in VAT from the nonobese CRC (N = 27) (**a**), nonobese control (N = 53) (**b**), overweight/obese CRC (N = 58) (**c**) overweight/obese control (N = 81) (**d**) groups. the square that are not crossed out with a cross are significant (* *p* < 0.05). Abbreviations: C/EBP-α: CCAAT/enhancer-binding protein type alpha; PPAR-γ2: peroxisome proliferator-activated receptor gamma; PGC-1α: peroxisome proliferator-activated receptor gamma coactivator 1-α; NF-κB: nuclear factor κ-light-chain-enhancer of activated B cells; VAT: Visceral adipose tissue.

We next investigated the relationship between gene expression and methylation status of C/EBP-α, PPAR-γ, PGC-1α, and NF-κB in VAT and anthropometric and biochemical variables in the population study. For that, a Pearson’s correlation analysis was performed, and results are summarized in Figure 4.

In nonobese subjects, C/EBP-α expression seems to be correlated with adipogenic transcription factors (significant positive correlation with PPAR-γ and PGC-1α (*p* < 0.05)), while in nonobese CRC patients, C/EBP-α expression was more correlated with glucose (significant correlation with homeostasis model assessment of insulin resistance (HOMA-IR) and glucose levels (*p* < 0.05)) and lipid metabolism (positive trend with triglycerides and negative correlation with HDL (*p* < 0.05)). Additionally, in overweight/obese subjects, C/EBP-α expression remain correlated with adipogenic and inflammatory factors (negatively correlated with PPAR-γ and NF-κB expression and positively correlated with PPAR-γ methylation (*p* < 0.05)), while in overweight/obese CRC patients, C/EBP-α expression also remains correlated with glucose metabolism (HOMA-IR and insulin levels (*p* < 0.05)). In addition, C/EBP-α methylation showed a significant positive correlation with PGC-1α gene (PGC-1α methylation in overweight/obese CRC patients and PGC-1α expression in overweight/obese subjects (*p* < 0.05)).

PPAR-γ expression seems to have a close relationship with overweight/obese subjects, since it was correlated with adipogenic and inflammatory factors (positively correlated with PGC-1α and NF-κB expression and negatively correlated with PPAR-γ methylation (*p* < 0.05)), while PPAR-γ methylation was more related to anthropometric data (waist circumference) in nonobese subjects and nonobese CRC patients.

The same trend was observed in PGC-1α expression. In overweight/obese subjects, PGC-1α expression and methylation showed strong correlation with adipogenic and inflammatory factors (PGC-1α expression was positively correlated with PPAR-γ and NF-κB expression and C/EBP-α methylation (*p* < 0.05), and PGC-1α methylation was positively correlated with NF-κB and C/EBP-α methylation (*p* < 0.05)). NF-κB expression also showed strong correlation with adipogenic factors (positively correlated with C/EBP-α, PPAR-γ, and PGC-1α expression (*p* < 0.05)) in overweight/obese individuals.

### 2.5. Gene Expression and Methylation Status in VAT Are Predictors of CRC

Given that NF-κB and C/EBP-α gene expression and NF-κB methylation in VAT were correlated with CRC, we performed a multiple linear regression analysis to test the predictive value of NF-κB and C/EBP-α gene expression and NF-κB methylation in VAT as s risk marker for developing CRC. Thus, CRC was taken as a dependent variable and corrected by variables strongly related to CRC as age, sex, and BMI. This model reached a strong significance, showing that CRC was greatly explained by NF-κB gene expression and methylation. In this model, these variables could explain up to 55% of the CRC variability in our study population (Table 2).

## 3. Discussion

The exact mechanisms that link CRC with obesity are not fully understood yet. It is well known that obesity associates with CRC through a number of metabolic abnormalities and that several of these have been linked to an increased risk of CRC. The role of the adipose tissue in tumor initiation, growth, and metastasis is considered to be a relatively new area of investigation. However, the characteristics of the tissue’s proximal and distal microenvironments are proposed to play an integral role in supporting the proliferation of cancer cells [27,28]. Therefore, it is important to understand the complex mutual relationships between colon cancer cells and adipose tissue and how these interactions may alter colon cancer metabolism and promote carcinogenesis. As already reported, adipose tissue in the obese state is characterized by chronic inflammation and enriched proportions of inflammatory cells. These inflammatory cells, together with the altered resident adipocytes, secrete significant amounts of adipokines and other cytokines and activate others transcription factors, which have been implicated in the promotion of tumor growth [29].

In this study, we analyzed the expression and methylation status of C/EBP-α in VAT, the context of obesity and CRC. We found that the expression profile of C/EBP-α was decreased in VAT from overweight/obese CRC patients in comparison with overweight/obese subjects. The role of C/EBP-α in adipose tissue is well established, since it induces adipogenesis through PPAR-γ in an unified pathway, and in cell culture, C/EBPα is sufficient to trigger differentiation of pre-adipocytes into mature adipocytes [30,31]. However, the role of C/EBP-α in CRC remains controversial. A study showed that C/EBPα was upregulated in CRC tumor, promoted tumor growth both in vitro and in vivo, and acted as an oncogene in CRC, suggesting that C/EBP-α seems to play a promoting role in colorectal tumorigenesis and progression [11], while other study suggested C/EBP-α as a tumor suppressor gene because downregulation of C/EBP-α was presented in various tumors and showed correlation with progression, poor prognosis, and tumor size [9]. In any case, all these studies pointed out the critical role of C/EBP-α in cancer, which plays a crucial function in the regulation of mitotic growth arrest and differentiation in numerous cell types, including tumoral cells [9,32,33,34].

In our study, the expression of C/EBP-α in VAT from overweight/obese CRC (decreased expression) patients seems to be in line with Tseng and colleagues, suggesting that C/EBP-α could be a tumor suppressor gene in VAT. These results suggest the importance of VAT as a metabolic tissue in CRC and how a dysfunctional role of VAT could be related to CRC development through an altered expression profile of potential genes considered such as C/EBP-α, suggesting that an increased BMI may alter the expression of C/EBP-α in CRC patients [9].

On the other hand, our results showed that C/EBP-α expression was correlated with adipogenic and inflammatory factors in the absence of CRC while it was more correlated with glucose and lipid metabolism in the presence of CRC. A study conducted by Wu and colleagues, reported that, in cell culture, C/EBPα^−/−^ adipocytes are normal in almost every way with the important exception that they do not show insulin sensitivity [35]. Another study pointed out that C/EBP-α in adipose tissue regulates genes involved in lipid and glucose metabolism such as adiponectin, hexokinase 2, or lipoprotein lipase [36], suggesting that C/EBP-α may also have a crucial role in glucose and lipid metabolism. Our results suggest that C/EBP-α in CRC may have a more contributing effect in glucose and lipid metabolism rather than an interaction with adipogenic and inflammatory factors, as suggested by our results.

The function of PPAR-γ in adipose tissue is related to the regulation of essential aspects of biology from development to metabolism. PPAR-γ is considered the master regulator of adipogenesis, since it is involved in adipocyte differentiation, regulation of insulin sensitivity, lipogenesis, and adipocyte survival and function [37,38]. PPAR-γ is also considered as a tumor suppressor gene. Previous studies shed light to a protective role of PPAR-γ in CRC. These studies showed that PPAR-γ was directly implicated in the upregulation of others tumor suppressor genes, as Phosphatase And Tensin Homolog (PTEN), and downregulation of the antiapoptotic protein Bcl-2 [39,40]. PPAR-γ also reduced angiogenesis processes and interference in the APC/β-catenin signaling pathways, which were crucial in carcinogenesis of CRC. These data combined to firmly support the potential role of PPAR-γ as CRC suppressor [41]. However, in our results, no significant difference was found in PPAR-γ expression in adipose tissue in the presence of CRC, although we found a negative correlation between the PPAR-γ gene expression and methylation in the overweight/obesity state (overweight/obese subjects and overweight/obese CRC patients), suggesting that PPAR-γ expression and methylation in adipose tissue seem to be more important in the context of the obesity rather than CRC. The same trend was observed in PPAR-γ expression and its correlation with overweight/obesity state. Only in overweight/obese subjects, PPAR-γ expression was correlated with adipogenic and inflammatory transcription factors and PPAR-γ methylation was related to anthropometric variables, confirming the role of PPAR-γ in VAT in obesity state.

PGC-1α interacts with a broad range of transcription factors, such as PPAR-γ, that are involved in a wide variety of biological responses, including adaptive thermogenesis, mitochondrial biogenesis, glucose/fatty acid metabolism, and tissue development [42], although in adipose tissue, PGC-1α seems to have more relationship with glucose and lipid metabolism, insulin sensitivity, insulin resistance, and type 2 diabetes [43,44,45]. However, several studies linked PGC-1α to cancer, which has a biphasic role, acting both as tumor suppressor and tumor promoter gene [15,46]. PGC-1α expression levels are not dependent on cancer type. Various studies showed different expression levels of PGC-1α in the same cancer type, since PGC-1α expression profile (low or high expression) is not characteristic of a type of cancer from a specific organ or tissues [20]. For instance, PGC-1α was found overexpressed in human colon cell lines during tumor developments and progression [16], while another study pointed out a decreased expression in colon-derived tumor tissue compared to normal adjacent tissue [47]. In our results, PGC-1α expression was increased in VAT from CRC patients independently of BMI, suggesting that PGC-1α in adipose tissue could increase the risk of CRC, acting as a tumor promoter gene. Nevertheless, PGC-1α expression was more correlated with adipogenic and inflammatory factors only in overweight/obese individuals, especially with PPAR-γ and NF-κB expression, which is in line with previous studies in the development of adipose tissue [42]. Indeed, a previous study reported that PGC-1α directly interact with NF-κB, which promotes the pro-inflammatory state, a typical phenotype in obese subjects [48].

After all, PPAR-γ and PGC-1α are emerging transcription factors that are related to CRC. They are also an attractive topic to amplify our understanding of how VAT could be important in CRC. Despite both proteins discovered acting together, their functional actions were not related in cancer. The mechanism of PPAR-γ and PGC-1α in cancer is still contradictory with dual effects, of which the molecular interactions of PPARγ and PGC-1α with other transcriptional factor are necessary to be further investigated to clarify the role of PPARγ and PGC-1α in CRC [46].

The chronic activation of NF-κB in adipose tissue augments secretion of a wide range of adipokines, cytokines, and inflammatory factors such as MCP-1, IL-6, and TNF-α, driving a low-grade inflammation state [49,50]. This state has been largely linked to an increased risk of CRC [51]. The role of NF-κB in CRC is largely documented, of which there are more evidences between inflammation and tumorigenesis [52]. Constitutive NF-κB activation is able to induce several cellular responses and physiological alterations in many types of cancer cells. Constitutively activated NF-κB transcription factors have been related to typical aspects of tumorigenesis, including promoting cancer-cell proliferation, preventing apoptosis, and increasing a tumor’s angiogenic and metastatic phenotype [21].

In our study, our results showed that NF-κB expression in VAT was increased in CRC patients independently of the obesity state. Thus, it could suggest the role of a dysfunctional adipose tissue to promote a pro-inflammatory state and, therefore, to increase the risk of CRC. Indeed, a study was in line with our results, which reported that NF-κB in VAT was highly expressed in CRC patients but without taking the effect of the BMI, as we showed in our study [6]. However, in our results, NF-κB expression was more correlated with adipogenic factors only in overweight/obese individuals. Indeed, a tight interaction has been reported between NF-κB and PPAR-γ, of which PPAR-γ could inhibit the action of NF-κB [53].

Most studies showed that adipogenic and inflammatory metabolism are altered in obesity and cancer. Therefore, the role of adipose tissue metabolism on carcinogenesis is less well defined and more evidence is coming out to solve multiple missing points. Our results suggests that C/EBP-α, PGC-1α, and NF-κB are important to consider as potential biomarkers in the context of obesity and CRC, as their expression and methylation status could provide a new insight into how VAT is implicated in obesity-related CRC.

## 4. Materials and Methods

### 4.1. Study Design and Subjects

We included in this study a total of 219 participants recruited at the “Virgen de la Victoria” University Hospital of Malaga (Malaga, Spain). The subjects were subdivided into four group based in their BMI and the presence of CRC, of which 53 of them were nonobese control individuals (BMI < 25 Kg/m^2^), 27 were nonobese CRC individuals, 81 were overweight/obese (BMI ≥ 25 Kg/m^2^) control subjects, and 58 overweight/obese CRC individuals. The weights of all participants were stable in the last 6 months prior to participation in the study. The exclusion criteria were patients who had cardiovascular disease, arthritis, diabetes mellitus, acute or chronic inflammatory diseases, or renal and infectious diseases, and patients who had received treatment that alter the lipid or glucose profile or who consumed >20 g of ethanol/day. All participants gave their written informed consent, and the study was reviewed, approved by the Ethics Committees of “Virgen de la Victoria” University Hospital (Málaga, Spain), and performed in accordance with the “Declaration of Helsinki” (0311/PI7). Epiploic visceral adipose tissue (VAT) tissue was obtained during surgery. All tissue was frozen in liquid nitrogen and maintained at −80 °C until analysis.

### 4.2. Biochemical Determination

Blood samples from all participants were obtained after an overnight fast and before surgery. Serum was separated by centrifugation for 15 min at 4000 rpm at 4 °C and frozen at −80 °C. Serum Glucose, triglycerides, total cholesterol, high-density lipoprotein cholesterol (HDL), and alkaline phosphatase measurements were carried out using a Dimension Autoanalyzer (Dade Behring Inc., Deerfield, IL, USA). The insulin levels were performed using an immunoradiometric assay (BioSource International, Camarillo, CA, USA). Calculated values for low-density lipoprotein cholesterol (LDL) was obtained by the Friedewald formula [54]. The HOMA-IR was obtained by the following equation: HOMA-IR = fasting insulin (μIU/mL) × fasting glucose (mmol/L)/22.5 [55].

### 4.3. Total RNA Extraction from VAT and Real-Time Quantitative PCR

Total RNA isolation from VAT was isolated using RNeasy Lipid Tissue Mini Kit according to the manufacturer´s instructions (Qiagen GmbH, Hilden, Germany). To generate first-strand cDNA synthesis, we used 1 μg of total RNA, Moloney Murine Leukemia Virus reverse transcriptase, and random hexamers primers (Roche Diagnostic, Rotkreuz, Switzerland) as indicated by the manufacturer. We used commercially available TaqMan primer/probe mix (Integrated DNA Technologies Inc, Madrid, Spain) for the quantification of C/EBP-α (Hs.PT.58.3605816, RefSeq. NM_001079533), PPAR-γ (Hs.PT.58.4423955, RefSeq. NM_005036), PGC-1α (Hs.PT.58.4461873, RefSeq. NM_002630), NF-κB (Hs00765730_m1, RefSeq. NM_001165412.1 and NM_003998.3), DNTM3a (Hs.PT.58.28037916, RefSeq. NM_001130823), and PPIA (4326316E, RefSeq. NM_021130.3), used as endogenous controls for the target genes. Gene expression was carried out by real-time PCR using an Applied Biosystems 7500 Fast Real-Time PCR System (Applied Biosystems, Darmstadt, Germany) with TaqMan technology. Changes of gene expression were calculated by the 2−ΔΔCt method [56]. The results of the expression were represented as the target gene/PPIA ratio.

### 4.4. Pyrosequencing Analysis

DNA isolation from VAT was performed using Qiamp DNA Tissue Kit following the manufacturer´s instructions (Qiagen GmbH, Hilden, Germany). We used 2 μg of genomic isolated for bisulfite conversion. After that, PCR amplification was performed to amplify the promoter sequence for C/EBP, PPAR-γ, PGC-1α, and NF-κB. We used a premade Pyromark CpG assay for C/EBP, PPAR-γ, PGC-1α, and NF-κB. Finally, the sequencing process of the PCR products was performed using the PyroMarkTMQ96 ID Pyrosequencing System (Qiagen GmbH, Hilden, Germany). The level of methylation was presented as the percentage of methylated cytosine over the sum of methylated and unmethylated cytosines. We included unmethylated and methylated DNA as controls in each run (New England Biolabs, Ipswich, MA, USA). Inter-assay precision (%CV) was <2.5%; intra-assay (%CV) was <1.0%.

### 4.5. Statistical Analysis

The descriptive data of participants are shown as mean ± standard deviation (SD) for continuous measures and as numbers and percentages for categorical variables. Due to the sample size and the non-normal distribution of the expression and average methylation variables, we used nonparametric statistical Wilcoxon tests to assess differences between CRC/control groups within each BMI classification in terms of gene expression and average methylation. A *t*-test or Wilcoxon test was applied according to normality to anthropometric and biochemical variables. We used Pearson’s correlation coefficient to evaluate whether the expression and/or the methylation of the different genes correlated between them and with anthropometric and biochemical parameters. Analyses were performed using R v.3.5.1 software and significance was set at *p* < 0.05.

## 5. Conclusions

In conclusion, we provide data supporting the idea that adipogenic and inflammatory transcriptions factors in VAT DNA are related to the etiology of obesity and CRC. We show that C/EBP-α expression was decreased in overweight/obese CRC patients and that PGC-1α and NF-κB expression was increased in CRC patients. In addition, C/EBP-α expression was correlated with glucose and lipid metabolism in CRC patients, while PGC-1α and NF-κB have more relationship in overweight/obese subjects by correlating with adipogenic and inflammatory factors. Our study shows the importance of adipose tissue for a better understanding of the close relationship between obesity, adipose tissue, and risk of CRC, which could lead to new biomarkers strategies.

## Figures and Tables

**Table 1:** Baseline and general characteristics of the participants.

Variables	Nonobese Control	Nonobese CRC	Overweight/Obese Control	Overweight/Obese CRC
N	53	27	81	58
Male/female, sex (%)	22/31 (41.5/48.5)	19/8 (70.40/29.60) ^a^	36/45 (44.4/55.6)	42/16 (72.40/27.60) ^b^
Age (years)	47.94 ± 13.63	66.7 ± 12.98 ^a^	54.78 ± 14.43	68.22 ± 9.27 ^b^
Weight (kg)	65.30 ± 9.58	65.43 ± 7.93	78.39 ± 12.34	78.19 ± 11.49
Waist (cm)	83.04 ± 9.20	87.57 ± 11.27	99.86 ± 10.95	101.84 ± 12.72
BMI (kg/m^2^)	22.97 ± 1.73	23.19 ± 1.61	30.02 ± 3.59	29.09 ± 3.34
Glucose (mg/dL)	91.97 ± 14.14	133.35 ± 79.53	108.08 ± 28.13	119.36 ± 38.86
Insulin (µIU/mL)	7.30 ± 5.47	9.01 ± 6.12 ^a^	10.5 ± 5.68	7.30 ± 0.95 ^b^
Hb1Ac (%)	5.56 ± 0.52	5.78 ± 0.84	5.84 ± 1.23	5.93 ± 0.95
HOMA-IR	2.11 ± 1.66	1.36 ± 1.28	1.98 ± 1.26	1.08 ± 1.26
Cholesterol (mg/dL)	197.92 ± 29.94	178.54 ± 45.76	211.89 ± 39.89	170.43 ± 38.89 ^b^
Triglycerides(mg/dL)	108.14 ± 72.45	142.69 ± 74.62	131.90 ± 54.35	170.00 ± 79.35 ^b^
HDL (mg/dL)	54.84 ± 14.46	43.77 ± 14.04 ^a^	53.94 ± 12.77	40.34 ± 13.83 ^b^
LDL (mg/dL)	122.39 ± 24.58	108.41 ± 35.68	132.54 ± 32.31	101.07 ± 32.31 ^b^
Alkaline phosphatase (U/L)	62.06 ± 25.79	63.62 ± 24.69	71.70 ± 19.92	71.97 ± 29.63

Results are presented as means ± SD. ^a^ Significant difference between nonobese (BMI < 25 kg/m^2^) control and nonobese CRC; ^b^ Significant difference between overweight/obese (BMI ≥ 25 kg/m^2^) control and overweight/obese CRC patients according Wilcoxon test and Chi squared test for gender variables expressed as percentage (*p* < 0.05). Abbreviations: CRC: Colorectal cancer; BMI: Body Mass Index; HbA1c: Glycated hemoglobin; HOMA-IR: Homeostasis model assessment of insulin resistance; HDL: High density lipoprotein; LDL: Low density lipoprotein.

**Table 2:** Linear regression analysis of gene expression and methylation status in VAT and CRC.

Variables	β (SE)	*p*	CI (95%)
Age	0.013 (0.002)	0.000	0.007–0.018
Sex	−0.191 (0.080)	0.047	−0.350–(−0.032)
BMI	0.001 (0.005)	0.856	−0.009–0.012
C/EBP-α expression	0.510 (0.260)	0.054	0.008–1.028
NF-κB expression	3.687 (0.641)	0.000	2.410–4.963
NF-κB methylation	−0.087 (0.030)	0.005	−0.147–(−0.026)

Results are presented as β (SD). Abbreviations: CRC: Colorectal cancer; BMI: Body Mass Index; C/EBP-α: CCAAT/enhancer-binding protein type alpha; PPAR-γ2: peroxisome proliferator-activated receptor gamma; PGC-1α: peroxisome proliferator-activated receptor gamma coactivator 1-α; NF-κB: nuclear factor κ-light-chain-enhancer of activated B cells; VAT: Visceral adipose tissue.

## Supplementary Materials

The following are available online at https://www.mdpi.com/2072-6694/11/11/1629/s1, Figure S1: Gene expression and methylation analyses of CCAAT-enhancer binding protein type alpha (C/EBP-α) and peroxisome proliferator-activated receptor gamma (PPAR-γ) in visceral adipose tissue (VAT). Figure S2: Gene expression and methylation analyses of PGC-1α and NF-κB in VAT.
