# Supplementary material for: The Expression/Methylation Profile of Adipogenic and Inflammatory Transcription Factors in Adipose Tissue Are Linked to Obesity-Related Colorectal Cancer
Source: Cancers (Basel). 2019 Oct 24;11(11):1629. doi: 10.3390/cancers11111629 (PMC6893417; doi:10.3390/cancers11111629)
Supplement: Supplementary file 1 [file cancers-11-01629-s001.pdf]

Supplementary material

The Expression/Methylation Profile of Adipogenic and Inflammatory Transcription Factors in Adipose Tissue Are Linked to Obesity-Related Colorectal Cancer

Hatim Boughanem, Amanda Cabrera-Mulero, Pablo Hernández-Alonso, Borja Bandera-Merchán, Alberto Tinahones, Francisco José Tinahones, Sonsoles Morcillo and Manuel Macias-Gonzalez

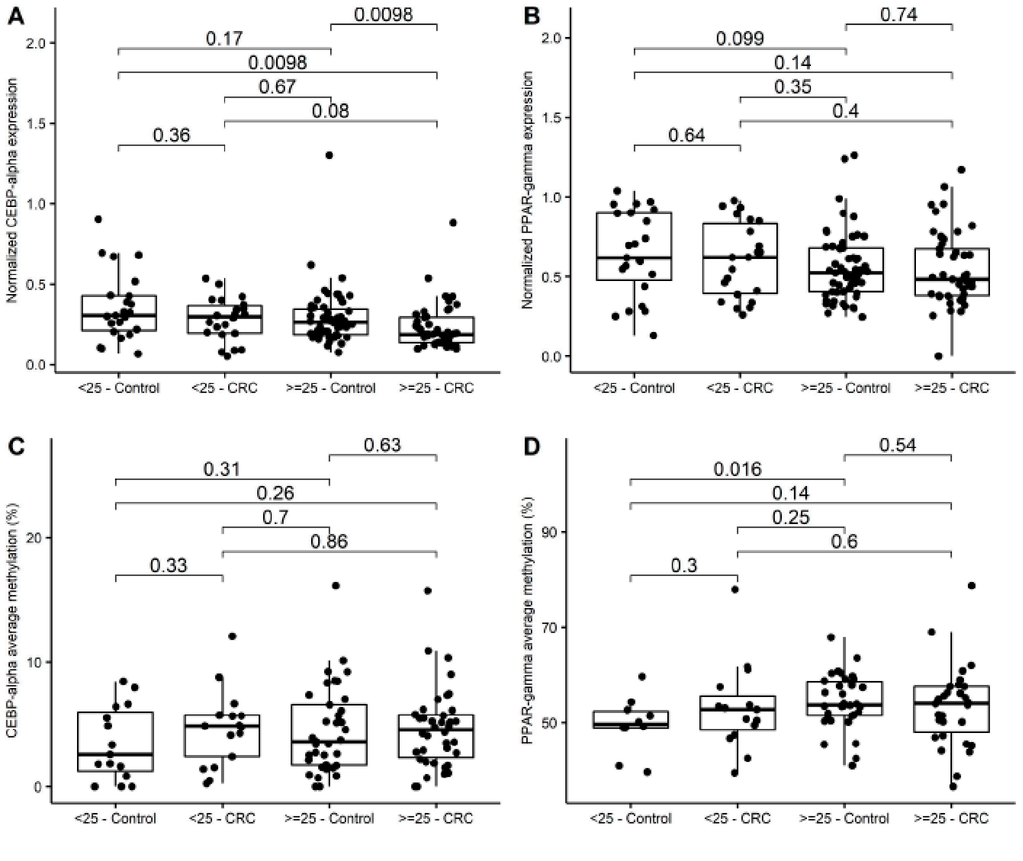

Figure S1. Gene expression and methylation analyses of CCAAT-enhancer binding protein type alpha (C/EBP-α) and peroxisome proliferator-activated receptor gamma (PPAR-γ) in visceral adipose tissue (VAT): Quantitative RT-PCR of C/EBP-α (**a**) and PPAR-γ (**b**) expression analyses and methylation analyses at specific CpG dinucleotides for C/EBP-α (**c**) and PPAR-γ (**d**) promoters was used to compare the nonobese control (N = 53), nonobese CRC (N = 27), overweight/obese control (N = 81), and overweight/obese CRC groups (N = 58). The mRNA expression of C/EBP-α and PPAR-γ were normalized to PPIA expression. The results are given as the mRNA relative mean expression ± SD and methylation average ± SD. Significant differences between the means of the different groups of subjects was performed according to the Wilcoxon test ($p < 0.05$). Abbreviations: C/EBP-α: CCAAT/enhancer-binding protein type alpha; PPAR-γ2: peroxisome proliferator-activated receptor gamma; VAT: Visceral adipose tissue.

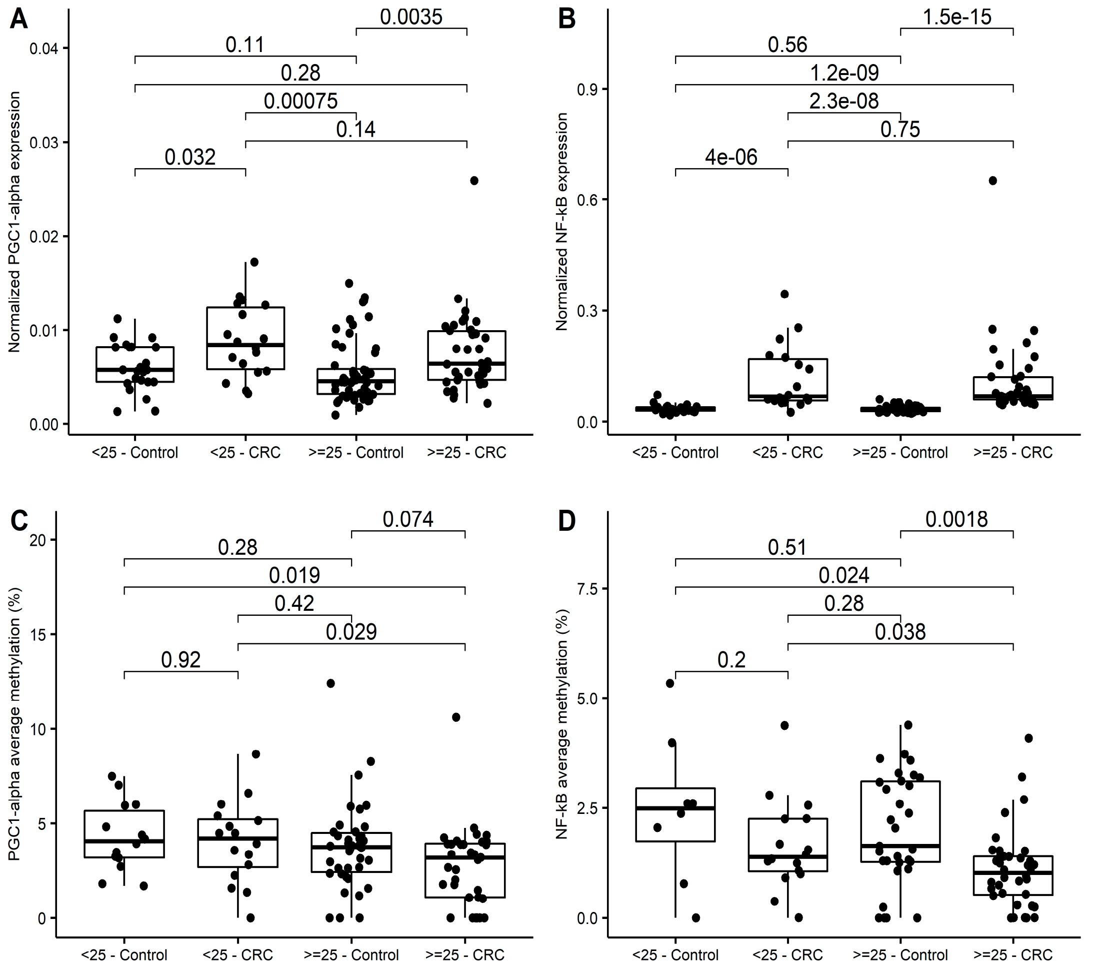

Figure S2. Gene expression and methylation analyses of PGC-1α and NF-κB in VAT: Quantitative RT-PCR of PGC-1α (**a**) and NF-κB (**b**) expression analyses and methylation analyses at specific CpG dinucleotides for PGC-1α (**c**) and NF-κB (**d**) promoters was used to compare the nonobese control (N = 53), nonobese CRC (N = 27), overweight/obese control (N = 81), and overweight/obese CRC groups (N = 58). The mRNA expression of PGC-1α and NF-κB was normalized to the PPIA expression. The results are given as the mRNA relative mean expression ± SD and methylation average ± SD. Significant differences between the means of the different groups of subjects was performed according to the Wilcoxon test ($p < 0.05$). Abbreviations: PGC-1*α*: peroxisome proliferator-activated receptor gamma coactivator 1-*α*; *NF-κ*B*:* nuclear factor κ-light-chain-enhancer of activated B cells; VAT: Visceral adipose tissue.

© 2019 by the authors. Licensee MDPI, Basel, Switzerland. This article is an open access article distributed under the terms and conditions of the Creative Commons Attribution (CC BY) license (http://creativecommons.org/licenses/by/4.0/).
